# Phosphate-containing dialysis solution prevents hypophosphatemia during continuous renal replacement therapy

**DOI:** 10.1111/j.1399-6576.2010.02338.x

**Published:** 2011-01

**Authors:** M BROMAN, O CARLSSON, H FRIBERG, A WIESLANDER, G GODALY

**Affiliations:** 1 Department of Anaesthesiology and Intensive Care, Lund University HospitalLund, Sweden; 2 Gambro Lundia ABLund, Sweden

## Abstract

**Background:**

Hypophosphatemia occurs in up to 80% of the patients during continuous renal replacement therapy (CRRT). Phosphate supplementation is time-consuming and the phosphate level might be dangerously low before normophosphatemia is re-established. This study evaluated the possibility to prevent hypophosphatemia during CRRT treatment by using a new commercially available phosphate-containing dialysis fluid.

**Methods:**

Forty-two heterogeneous intensive care unit patients, admitted between January 2007 and July 2008, undergoing hemodiafiltration, were treated with a new Gambro dialysis solution with 1.2 mM phosphate (Phoxilium) or with standard medical treatment (Hemosol B0). The patients were divided into three groups: group 1 (*n*=14) receiving standard medical treatment and intravenous phosphate supplementation as required, group 2 (*n*=14) receiving the phosphate solution as dialysate solution and Hemosol B0 as replacement solution and group 3 (*n*=14) receiving the phosphate-containing solution as both dialysate and replacement solutions.

**Results:**

Standard medical treatment resulted in hypophosphatemia in 11 of 14 of the patients (group 1) compared with five of 14 in the patients receiving phosphate solution as the dialysate solution and Hemosol B0 as the replacement solution (group 2). Patients treated with the phosphate-containing dialysis solution (group 3) experienced stable serum phosphate levels throughout the study. Potassium, ionized calcium, magnesium, pH, *p*CO_2_ and bicarbonate remained unchanged throughout the study.

**Conclusion:**

The new phosphate-containing replacement and dialysis solution reduces the variability of serum phosphate levels during CRRT and eliminates the incidence of hypophosphatemia.

The majority of patients on continuous renal replacement therapy (CRRT) will require phosphate supplementation shortly after CRRT initiation.^1^ One reason is that critically ill patients present several conditions predisposing hypophosphatemia such as sepsis, alcohol withdrawal, malnutrition, catecholamines, intravenous glucose infusion, hyperventilation, diuretics and rhabdomyolysis.^2^–^4^ Another reason is the CRRT technique that achieves high clearance of small solutes, such as phosphate.^5^–^9^ In addition, low serum phosphate levels may also occur in the setting of extracellular to intracellular shifts that occur with respiratory alkalosis, high blood concentrations of stress hormones (i.e., insulin, glucagon, adrenalin, cortisol) and with refeeding syndrome.

As phosphate is a constituent of enzymes and intermediate phosphorylated compounds, it plays a key role in cellular metabolism and is essential in several biological processes. Serum phosphate concentration is maintained between 0.81 and 1.45 mmol/l. By convention, hypophosphatemia is often graded as mild (<0.81 mmol/l), moderate (<0.61 mmol/l) and severe (<0.32 mmol/l). Severe hypophosphatemia has been linked to increased mortality in surgical intensive care patients^7^ and was recently shown to serve as an independent mortality predictor in sepsis.^10^ Symptoms of hypophosphatemia are usually only seen in patients with moderate or severe hypophosphatemia and include ventilatory muscle weakness, cardiac failure, insulin resistance, hemolysis, impaired platelet and white blood cell function, rhabdomyolysis, and, in rare cases, neurologic disorders.^3^,^11^–^17^ However, all these alternations have been shown to reverse by simply correcting the phosphate levels.^3^,^7^,^12^,^13^,^18^–^20^ Phosphate is supplemented intravenously in symptomatic patients, but phosphate has also been added directly to the dialysate and replacement fluids,^1^,^21^,^22^ with a risk of precipitation with calcium.

The development of many electrolyte disturbances in the intensive care unit (ICU) could be prevented by the use of better adapted dialysis fluids. This study evaluated the possibility to achieve and maintain a normal phosphate balance over time in patients on CRRT by using a new phosphate-containing dialysis fluid and replacement fluid.

## Methods

### Fluid composition and study design

We used a new phosphate-containing solution for dialysis that in addition to standard electrolytes also contains 4.0 mmol of potassium and 1.2 mmol of phosphate (Phoxilium, Gambro Lundia AB, Lund, Sweden, Table 1). As a control, we used our routine dialysis solution that does not contain phosphate (Hemosol B0, Gambro Lundia AB, Table 1). At our ICU at Lund University Hospital we applied three regimes, half a year each, for all patients requiring CRRT treatment. The treatment mode used was CVVHDF. During the first period (group 1), all the patients received dialysate solution and replacement solution that did not contain phosphate (Hemosol B0), during the next half year (group 2), all patients requiring CRRT treatment received the phosphate-containing solution as dialysis solution and a phosphate-free replacement solution (Hemosol B0) and finally during the last half year period (group 3), the patients received the phosphate-containing solution both as a dialysis solution and as a replacement solution. Blood sampling was performed according to normal routines at our department, but the physicians in charge continuously modified the CRRT treatment settings and the phosphate supplementation according to the patients' ongoing clinical needs. The physicians treating the patients did not have knowledge of the study setting during the treatments, but they were fully aware of the fluids and their contents.

**Table 1:** Fluid composition.

	Hemosol B0 (mmol/l)	Phosphate containingdialysis solution (mmol/l)
Bicarbonate	32	30
Lactate	3	0
Calcium	1.75	1.25
Magnesium	0.5	0.6
Potassium	0	4
Sodium	140	140
Phosphate	0	1.2
Chloride	109.5	115.9

### Patients and CRRT treatments

After acceptance by the Regional Ethical Review Board (DNR 570/2008), Lund University, Sweden, we evaluated retrospectively the first 14 patients who did not fulfill the exclusion criteria in each group, a total of 42 consecutive patients (Table 2). Patients were excluded if they had chronic kidney disease, if they had received intermittent dialysis before the ICU stay, if the CRRT treatment lasted <10 h or if they were under the age of 18 years. In all patients, the Gambro Prismaflex CRRT machine with the CVVHDF modality and a Hospal M100 filter (Hospal Industrie, Meyziew, France) was used. The blood flow, the dialysis fluid flow, the replacement fluid flow, the anticoagulation used and the fluid removal were set according to the patients' conditions and requirements. Of the replacement fluid, 500 ml/h was postfilter and the rest was prefilter in each treatment according to the general standard at the department. Intravenous phosphate addition was prescribed when serum phosphate was <0.8 mmol/l, also according to the general standard at the department. Nutrition was given only if the patients were hemodynamically stable as parenteral or enteral or both during the study period.

**Table 2:** Main diagnoses leading to intensive care of the study groups.

Main diagnoses	Group 1	Group 2	Group 3
Septic shock	5	8	7
Pneumonia	2	1	0
Cardiac insufficiency	0	0	3
Cardiac arrest	1	0	2
Major intoxication	1	0	1
Rupture of aortic aneurysm	0	0	2
Myocardial infarction	0	0	1
Cardiogenic shock	0	0	1
Sclerosis cholangitis	0	0	1
Unspecified respiratory failure	1	0	0
Obstructing malignant intestinal tumour	1	0	0
Epilepsy	1	0	0
Unspecified muscle disease	0	0	1

### Clinical parameters of the patients

All patients had regular measurements of serum sodium, potassium, ionized calcium, pH, *p*CO_2_ and bicarbonate either from an arterial line or from a central venous line before the start and regularly every fourth hour all through the CRRT treatment during 1, 2, 3, 4 and maximum 5 days. For phosphate and magnesium analyses, blood samples were taken at 5:00 hours in the morning and at 17:00 hours in the afternoon before the start and during the CRRT treatment days. Blood samples were analyzed at the hospital laboratory (Laboratory for Clinical Chemistry, Lund University Hospital, Lund, Sweden). The results are presented as means±SD for normal distributed data, median and range for remaining data.

In addition, baseline characteristics of study patients and delivered CRRT were registered (Table 3). Hypophosphatemia has been defined as condition where the serum phosphate level is <0.81 mmol/l.

**Table 3:** Baseline characteristics of study patients and delivered CRRT.

	Group 1	Group 2	Group 3
Demographics*			
Age (years)	63 (43–86)	68 (37–88)	67 (53–83)
Weight (kg)	80 (53–116)	86 (56–124)	79 (41–130)
Male sex, *n* (%)	7 (50)	7 (50)	5 (36)
APACHE II*	28.1 (19–44)	26 (13–36)	23.2 (15–29)
RIFLE score, points*,†	37	39	39
Delivered CRRT*			
CRRT treatment duration (h)	82 (17–278)	115 (37–207)	96 (29–210)
CRRT treatment mode	CVVHDF	CVVHDF	CVVHDF
Effluent flow (ml/kg/h) calculated on active treatment time*,‡	23.7 (13–45)	21.9 (15–30)	18 (10–20)
Proportion of ordered dose delivered*	85.3%	87.5%	91.8%
Anticoagulation*	None 3	None 2	None 2
	Heparine 9	Heparine 11	Heparine 10
	Prostacycline 1	Prostacycline 1	Prostacycline 2
Phosphate supplementation§ (mmol/CRRT treatment day)	11 (0–35)	3 (0–13)	0 (0)

* Differences between groups were not statistically significant. Numbers in parentheses are range, unless stated otherwise.

† We scored RIFLE-risk (R, risk; I, injury; F, failure; L, loss of kidney function; E, end-stage kidney disease) as 1, injury as 2, and failure as 3.

‡ The dialysis dose in CRRT is expressed in terms of ml of effluent (dialysate+ultrafiltrate) per kg of body weight (BW) per hour (ml/kg/h).^23^

§ Statistical differences were as follows: between groups 1 and 2 (*P*=0.046), between groups 1 and 3 (*P*≤0.001), and between groups 2 and 3 (*P*=0.003).

### Statistics

One-way repeated measures analysis of variance was used. The statistical program used was SigmaStat, version 3.5, for Windows XP. Differences were considered to be significant for *P*<0.05.

## Results

Main diagnoses leading to intensive care of the study groups, baseline characteristics, timing of initiation of CRRT, ultrafiltration rate, duration of the CRRT and anticoagulation were statistically similar between the three groups (Tables 2 and 3).

**Fig. 1:**
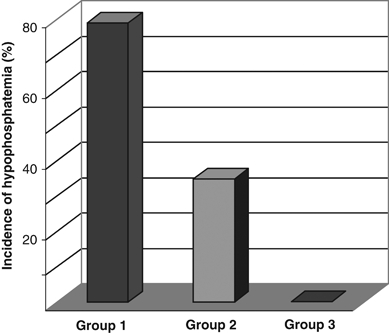
Events of hypophosphatemia during the continuous renal replacement therapy. Treatment with the phosphate-containing solution for dialysis resulted in no episodes of hypophosphatemia.

The incidence of hypophosphatemia occurred in 11 of 14 patients in group 1, where the patients did not receive the phosphate-containing dialysis solution, but received phosphate intravenously based on the serum phosphate values (Fig. 1). In group 2, where the patients received a phosphate-containing solution as the dialysis solution and a phosphate-free solution as the replacement solution, five of 14 patients had at least one episode of hypophosphatemia (Fig. 1). No episodes of hypophosphatemia were detected in group 3.

The serum phosphate level was 1.90 mmol/l at baseline and 0.99 mmol/l during CRRT treatment in group 1. In group 2, the corresponding values were 1.54 and 1.20 mmol/l, and in group 3, they were 1.83 and 1.43 mmol/l (Table 5). However, due to the simultaneous intake of enteral/parenteral solutions, two of 14 of the patients in group 3 had a temporary increase in serum phosphate (<1.9 mmol/l), but there were no cases of hyperphosphatemia that required a withdrawal of the phosphate-containing dialysis solution from the treatment.

**Table 5:** Serum values (mmol/l, kPa) during CRRT with the phosphate-containing dialysis fluid.

Group	Group 1		Group 2		Group 3	
	Before	During	Before	During	Before	During
Phosphate*	1.90	0.99	1.54	1.20	1.83	1.43
Ionized Ca†	1.10	1.24	1.13	1.19	1.11	1.15
pH‡	7.24	7.40	7.30	7.36	7.32	7.36
pCO_2_‡	5.59	5.27	5.81	5.77	6.51	5.27
Bicarbonate§	18	24	21	23	21	22

Mean values of each day of all the patients.

* *P*≤0.001 between the groups during the CRRT treatment.

† *P*=0.004 between the groups during the CRRT treatment.

‡ Differences between the groups were not statistically significant.

§ *P*=0.045 between the groups during the CRRT treatment.

Phosphate intake was from nutrition and from intravenous supplementation. The average phosphate input calculated from the total nutrition was 18 mmol/CRRT treatment/day for all groups. Short-acting insulin was administered as infusion in order to achieve a blood glucose level between 5 and 8 mmol/l. Phosphate was supplemented intravenously if serum phosphate declined to <0.80 mmol/l. In group 1, the average phosphate supplementation was 10 mmol/CRRT treatment/day. In group 2, the average supplementation was 5 mmol/CRRT treatment and day, and in group 3, there was no intravenous supplementation.

**Fig. 2:**
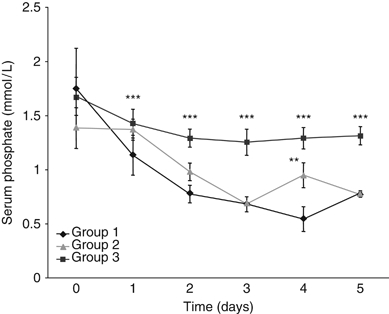
Serum phosphate levels during the continuous renal replacement therapy treatment. Group 1, receiving Hemosol B0 (diamond), group 2, receiving the phosphate-containing dialysis fluid as the dialysis fluid and Hemosol B0 as the replacement fluid (triangle), and group 3, receiving the phosphate-containing dialysis fluid (square). Groups 2 and 3 were statistically compared with group 1. *n*=14 patients/group, ^**^P<0.01, ^***^P<0.001.

There was a decline in phosphate levels during CRRT treatment in both groups 1 and 2. In group 1, serum phosphate declined constantly, although the patients received phosphate supplementation intravenously (Fig. 2). In group 2, the phosphate levels were unstable and reached a low level in the end of the study. In group 3, where the patients received a phosphate-containing solution both as the dialysis solution and the replacement solution, none of the patients had episodes of hypophosphatemia (Fig. 2). Phosphate remained stable in this patient population during the entire study.

For group 3, we also evaluated any adverse event in sodium, potassium and magnesium homeostasis. There was a significant increase in sodium (*P*≤0.001) and a slight increase in ionized calcium (*P*=0.029), whereas potassium and magnesium remained stable during the entire study as summarized in Table 4. A comparison between the study groups revealed that there was a significant difference in phosphate (*P*≤0.001), ionized calcium (*P*=0.004) and bicarbonate (*P*=0.045) between the groups (Table 5). The pH and *p*CO_2_ measurements were obtained at baseline and rapidly declined toward normal values after starting the CRRT treatment and remained essentially unchanged during the treatment in all groups (Table 4).

**Table 4:** Serum solute concentrations (mmol/l) during CRRT with the phosphate containing dialysis fluid for patients in group 3.

Day on CRRT	Before	1	2	3	4	5
Sodium*	134.0 ± 5	135.0 ± 3	135.2 ± 2	135.0 ± 2	135.7 ± 2	135.8 ± 3
Potassium†	4.06 ± 0.5	4.10 ± 0.4	4.14 ± 0.3	4.23 ± 0.3	4.39 ± 0.4	4.49 ± 0.5
Ionised calcium‡	1.11 ± 0.08	1.10 ± 0.05	1.15 ± 0.07	1.17 ± 0.06	1.16 ± 0.05	1.17 ± 0.06
Magnesium†	1.1 ± 0.4	1.0 ± 0.2	1.1 ± 0.2	0.7 ± 0.2	0.9 ± 0.1	1.0 ± 0.3

* *P*≤0.001 within the group.

† Differences within the group were not statistically significant.

‡ *P*=0.029 within the group.

## Discussion

This study demonstrates that the new phosphate-containing dialysis solution is safe, reduces the variability of serum phosphate levels during CRRT and reduces the incidence of hypophosphatemia.

Various protocols for intravenous phosphate supplementation have been studied in the last 30 years. Today, there is a trend toward the use of larger and faster boluses of phosphate because of high failure of repletion (20–70%) and the need for additional phosphate administration.^24^–^31^ Authors usually agree that larger amounts of phosphate are needed to correct total body deficit, but their fear of adverse reactions has prompted a restrained attitude. Recent studies on ICU patients confirm that a relatively rapid infusion of potassium phosphate is safe if baseline serum potassium is below 4.5 mmol/l.^1^ The infusion rate is thus consequently limited by the serum potassium levels, and also by serum calcium levels, as high phosphate serum levels could induce hypocalcemia, as phosphate could precipitate with calcium in blood vessels and tissues. Recently, phosphate has been injected into dialysis solutions during treatment, but there is a risk of precipitation.^1^,^21^,^22^ We evaluated whether phosphate-containing solutions for dialysis and replacement simplify phosphate replacement even further.

The frequency of phosphate disturbances in critically ill patients is high, although the figure varies considerably depending on the study, between 8.8% and 80%.^32^,^33^ In our study, the incidence of hypophosphatemia during CRRT was 79% in the control group. The incidence of hypophosphatemia was lower (35%) in patients receiving the phosphate-containing solution as the dialysis solution and Hemosol B0 as the replacement solution. There were no episodes of hypophosphatemia in patients receiving only the phosphate-containing solution, but there was an incidence of mild (>1.9 mmol/l) hyperphosphatemia in 14% of these patients. This is probably due to the simultaneous intake of nutritional support, but the phosphate levels were only marginally elevated without physiological consequences and did not require withdrawal of the phosphate-containing dialysis solution from the patient.

The amount of phosphate required to correct total body deficit is variable and depends on the cause of hypophosphatemia and the chronicity of the process.^3^,^11^,^24^ The many physiological rearrangements in ICU patients may explain the need for larger amounts required for repletion. In our study, phosphate addition was necessary for patients in groups 1 and 2. This is representative of our experience, where the majority of patients on CRRT require phosphate supplementation shortly after CRRT initiation despite nutritional support. Malnourished alcoholic patients have a larger deficit due to long-standing negative phosphate balance. Malnutrition induces a re-feeding syndrome that has been reported after only 48 h of fasting in the ICU.^2^ The distribution volume for phosphate might be increased,^24^ while insulin, carbohydrate, and catecholamine administration act to decrease the serum phosphate concentration.^2^,^3^,^11^,^34^

The incidence of hypophosphatemia in this study was 10% of the 42 patients before the start of CRRT. The incidence of hyperphosphatemia was 52%, which is slightly lower than that found in other studies, where 65–80% of patients present hyperphosphatemia before the start of the CRRT.^8^,^35^ Phosphate was within normal limits in 38% of patients even before the beginning of CRRT. The new dialysis fluid contains 30 mM bicarbonate compared with 32 mM in Hemosol B0. With prescribed CRRT clearances of ≥20 ml/kg/h, most acid–base disturbances can be managed with bicarbonate compositions of 25–35 mM.^36^ The normal serum range for bicarbonate is 22–30 mmol/l, which was achieved in patients in all groups, although the increase in group 1 was most significant. Ionized calcium increased in all groups during treatment time, but the increase was less marked in groups 2 and 3. There is a possibility that the slow increase in bicarbonate and ionized calcium could reflect the differences in the ion content between Hemosol B0 and the phosphate-containing dialysis fluid, although these differences could also be due to the limitations of this study. The fact that this was a retrospective observational study, without other intervention but for the dialysis solutions, means that bias due to the patients' illnesses and due to other treatments could affect ion concentrations. Even the phosphate concentration in parenteral and enteral nutrition cannot be completely excluded, although this would not alter the basic results of the study.

The next objective of the study was to evaluate whether the administered phosphate could alter calcium and potassium homeostasis. Phosphate could theoretically precipitate with calcium, which could lead to hypocalcemia in the patients. We found that the phosphate-containing dialysis fluid did not induce hypocalcemia during CRRT. Another important concern was whether this fluid would cause potassium overload, as potassium phosphate is used instead of sodium phosphate. We found that the phosphate-containing dialysis fluid did not induce hyperkalemia either. Potassium phosphate was favored over sodium phosphate because potassium usually has to be added to CRRT solutions. In addition, there were no disturbances of magnesium levels.

The present study shows that by using the phosphate-containing fluid both as the dialysis fluid and the replacement fluid, we could eliminate the episodes of hypophosphatemia. The new phosphate-containing dialysis fluid simplified the phosphate control and avoided rapid phosphate fluctuations with intravenous bolus administration.
